# Speculum-free portable preterm imaging system

**DOI:** 10.1117/1.JBO.29.5.052918

**Published:** 2024-01-27

**Authors:** Tananant Boonya-ananta, Mariacarla Gonzalez, Ajmal Ajmal, Vinh Nguyen Du Le, Edward DeHoog, Michael J. Paidas, Arumugam Jayakumar, Jessica C. Ramella-Roman

**Affiliations:** a Florida International University, Department of Biomedical Engineering, Miami, Florida, United States; b Optical Design and Engineering, Long Beach, California, United States; c Miller School of Medicine, Department of Obstetrics, Gynecology and Reproductive Sciences, Miami, Florida, United States; d Florida International University, Herbert Wertheim College of Medicine, Miami, Florida, United States

**Keywords:** polarized imaging, preterm labor, portable device, pregnancy, Mueller-matrix

## Abstract

**Significance:**

Preterm birth is defined as a birth before 37 weeks of gestation and is one of the leading contributors to infant mortality rates globally. Premature birth can lead to life-long developmental impairment for the child. Unfortunately, there is a significant lack of tools to diagnose preterm birth risk, which limits patient care and the development of new therapies.

**Aim:**

To develop a speculum-free, portable preterm imaging system (PPRIM) for cervical imaging; testing of the PPRIM system to resolve polarization properties of birefringent samples; and testing of the PPRIM under an IRB on healthy, non-pregnant volunteers for visualization and polarization analysis of cervical images.

**Approach:**

The PPRIM can perform 4×3 Mueller-matrix imaging to characterize the remodeling of the uterine cervix during pregnancy. The PPRIM is built with a polarized imaging probe and a flexible insertable sheath made with a compatible flexible rubber-like material to maximize comfort and ease of use.

**Results:**

The PPRIM device is developed to meet specific design specifications as a speculum-free, portable, and comfortable imaging system with polarized imaging capabilities. This system comprises a main imaging component and a flexible silicone inserter. The inserter is designed to maximize comfort and usability for the patient. The PPRIM shows high-resolution imaging capabilities at the 20 mm working distance and 25 mm circular field of view. The PPRIM demonstrates the ability to resolve birefringent sample orientation and full field capture of a healthy, non-pregnant cervix.

**Conclusion:**

The development of the PPRIM aims to improve access to the standard of care for women’s reproductive health using polarized Mueller-matrix imaging of the cervix and reduce infant and maternal mortality rates and better quality of life.

## 1 Introduction

The current global level of preterm infants born each year is 15 million. Among them, ∼1  million will die before the age of five due to complications that result from premature birth, which occurs before 37 weeks of gestation. Premature birth can lead to serious medical issues, including hearing, vision, and digestion defects; neurological disorders; long-term cognitive impairment; and respiratory disease.^1^^–^^3^ Recent studies estimate a $26 billion annual expenditure in the United States on health care for premature birth and associated congenital disabilities. Risk factors^4^ for preterm delivery include a history of preterm birth, a sonographic short cervix, multiple gestations, short intervals between pregnancies,^5^ hypertensive disorders, and antepartum hemorrhage.^6^^–^^12^

Ongoing high rates of preterm birth persist due to several challenges. First, the underlying cause of PTB remains unidentified in ∼60% of all spontaneous preterm births.^11^ Second, clinical tools for early and accurate spontaneous preterm birth risk detection are lacking.

During cervical extracellular matrix (ECM) remodeling, the cervix undergoes a transformation process from a closed and rigid to a flexible and compliant structure, allowing for the safe delivery of the fetus through to the vaginal canal. Since premature cervical remodeling resulting from structural defects, infection, or unknown causes precedes the onset of preterm birth, understanding the remodeling process in a term or preterm pregnancy is critical to defining therapeutic targets and developing clinical tools for early and accurate detection of preterm risk. Reorganization of the cervical ECM structure is a critical regulatory event that determines the mechanical function of the cervix. Fibrillar collagen is the major structural protein in the cervix that determines its load-bearing capabilities. As pregnancy progresses, the cervix changes the processing and assembly of fibrillar collagen that directly corresponds to mechanical strength. Several human and animal studies suggest that atypical changes in the ECM organization of the cervix precede spontaneous preterm birth.^13^^–^^17^ Therefore, assessing collagen architecture may be a valuable indicator for premature cervical remodeling.

Mounting evidence suggests that subtle changes in the stromal fibrous tissue throughout the cervix, particularly in the ectocervix accessible through the vaginal canal, can be observed during pregnancy.^18^^,^^19^

Mueller-matrix imaging (MMI) has been employed to image the pregnant cervix in both human and animal models, as this modality shows strong contrast to cervical fibrous tissue. In a preliminary pilot study, we demonstrated that collagen organization can be quantified *in vivo* through an order parameter K, which exhibits significant changes at a later stage of pregnancy.^18^^,^^20^^–^^22^ Others have utilized a similar approach and shown a substantial decrease in depolarization during pregnancy^19^

Several studies^23^^,^^24^ have reported that using a duckbill speculum, commonly employed for cervical inspection, Pap smear, or colposcopy, is a significant factor in women’s reluctance to undergo cervical screening. Successful strategies have been proposed to remove or limit the use of the speculum. For example, the callascope^23^^,^^25^^,^^26^ utilizes a combination of inserter and imaging systems to access the cervix, which patients have preferred for both self-use and clinical use.

In this paper, we leverage the work of Asiedu et al.^24^ to extend the concept of speculum-free imagery to an MMI system. Here, we describe a new portable preterm imaging system (PPRIM) capable of acquiring Mueller-matrix images of the cervix utilizing an inserter and an imager device.

## 2 Methods

**Fig. 1:**
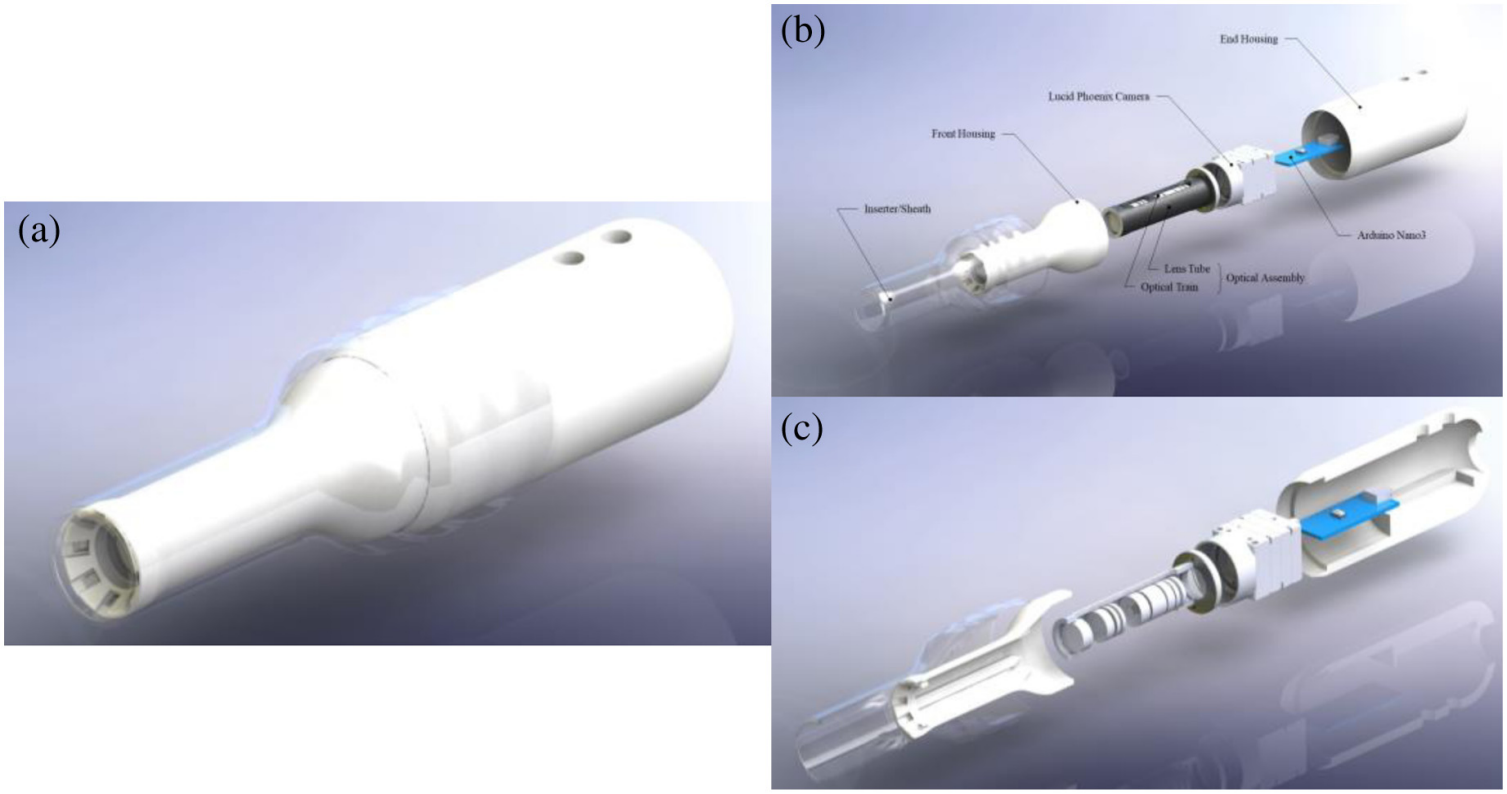
(a) Design assembly model render of PPRIM system; (b) exploded assembly of PPRIM indicating individual components; (c) exploded cross-section assembly of PPRIM, individual LED wire channels internal to the front housing, the lens optical train assembly and end housing internal features can be seen.

The main layout of the PPRIM is shown in Fig. 1. The system comprises two primary components: the inserter sheath and the housing assembly for the optics and electronics. Given that vaginal widths typically range from 48 to 63 mm, we designed the inserter with a diameter of ∼27.0  mm to enhance women’s comfort during the cervical inspection. Likewise, considering the varying length of the vaginal canal, which can be between 68.6 and 148.1 mm, we set the insertable length of the system at 65.0 mm.^27^

### 2.1 Mechanical Assembly

The structural portion of the system was designed using SolidWorks, a three-dimensional (3D) computer-aided design software. Stock models of the optical elements and camera were obtained from the manufacturers’ database. The design of the device housing and casing revolved around the SM05L30C Thorlabs lens tube and the Phoenix LUCID camera. A critical constraint parameter for the housing design was the maximum diameter and length of the inserted piece. The PPRIM was developed to accommodate these design parameters while integrating a spatially diverse illumination system.

**Fig. 2:**
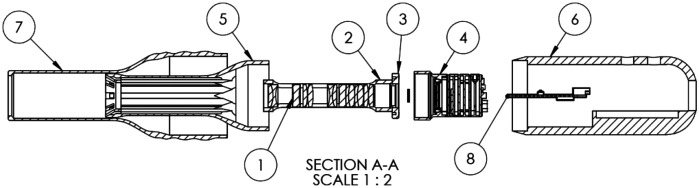
Explode assembly 2D drawing sectional view. Bill of materials can be seen in Table 1.

For prototyping, the housing was initially 3D printed using polylactic acid (PLA) on the MakerBot Method X. The final printed material, VisiJet M2R-WT (Rigid White), was produced using a Projet MJP 2500 printer from 3D Systems (Wilsonville, Oregon). VisiJet White material is rated for medical applications^28^ with tensile strength rated between 35 and 45 MPa and Young’s modulus between 1500 and 2000 MPa. The final design assembly is shown in Figs. 1 and 2.

**Table 1:** Bill of materials.

Item no.	Part number	Description	Quantity
1	—	Optical arrangement assembly	1
2	SM05L30C	Thorlabs lens tube	1
3	SM05A2	Thorlabs flange adaptor SM05 to C-Mount	1
4	PHX050S-P/Q	LUCID Phoenix polarized camera	1
5	—	Front-end housing case	1
6	—	Back-end housing case	1
7	—	Sheath	1
8	A000005	Arduino Nano3	1

**Fig. 3:**
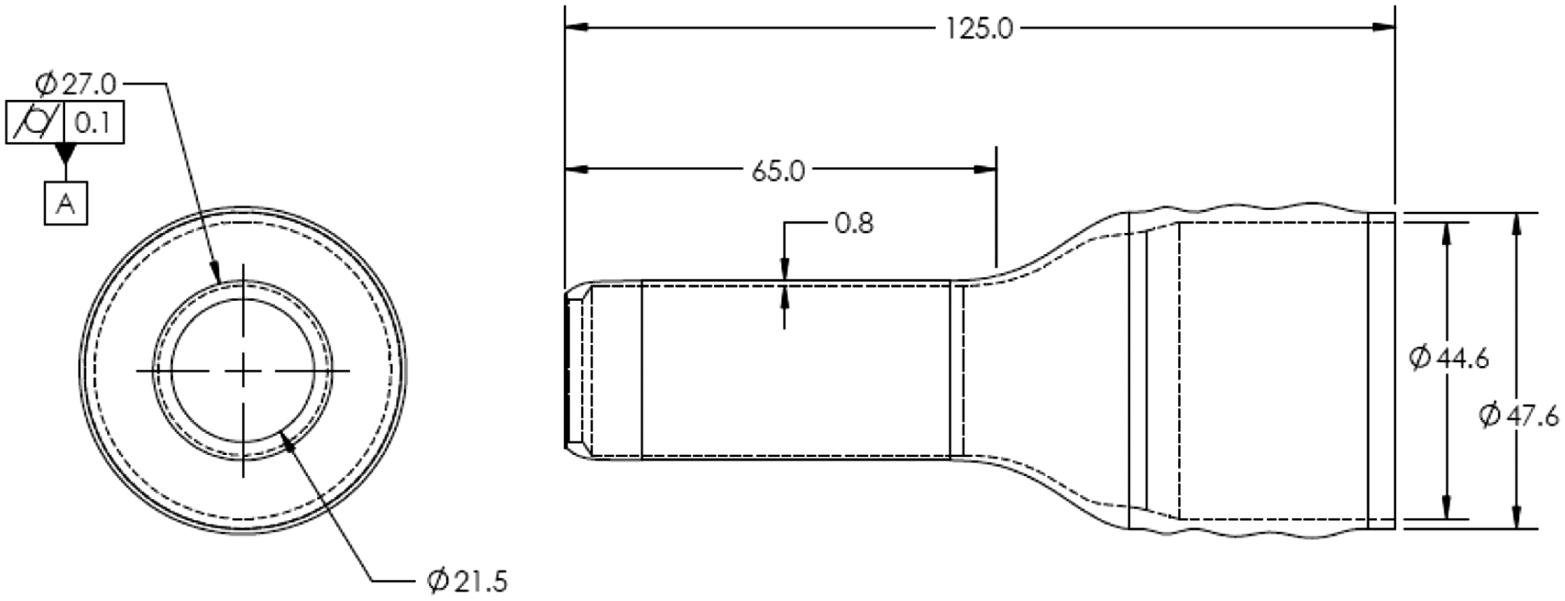
Inserter sheath dimension. All dimensions are in millimeters. The insertable length of the sheath is 65 mm, and the overall sheath length is 125 mm.

**Fig. 4:**
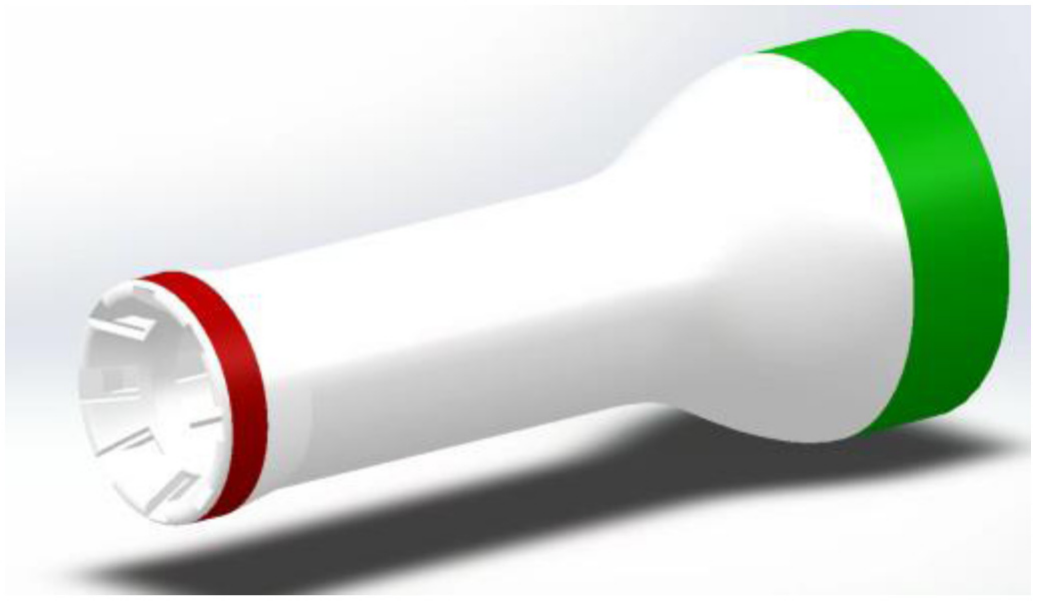
Overall dimensions of front housing with highlighted critical surfaces/features for design parameters. The red surface is critical for interface with the inserter sheath for a smooth insertion interface. The green surface is controlled for fitting into the back-end housing.

**Fig. 5:**
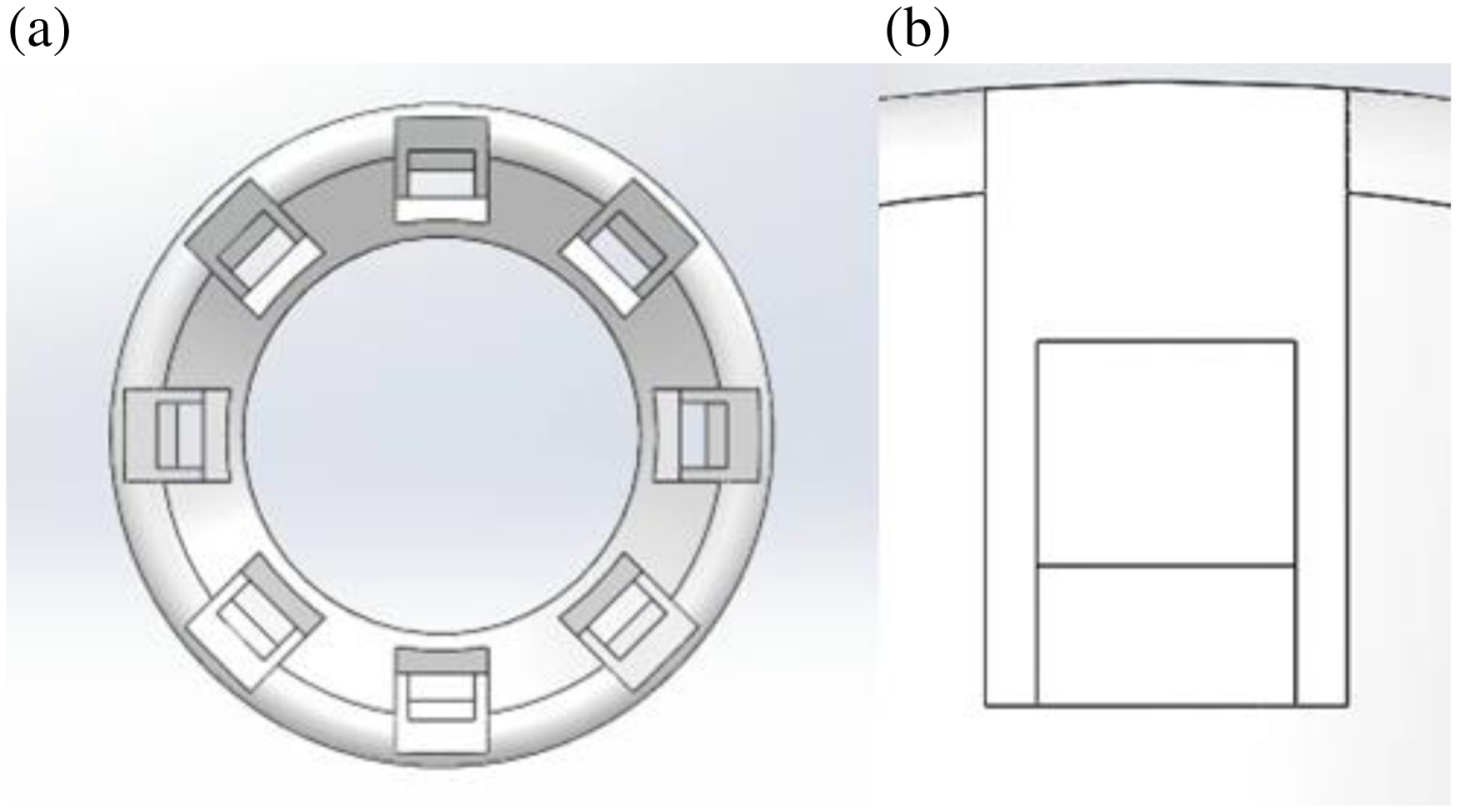
Front-end view of illumination and polarization components. (a) Model front view and (b) magnified single illumination pocket.

Figure 2 depicts the exploded assembly, providing a longitudinal cross-sectional view of the internal components. The design parameter of the main housing fits the lens tube in the center with eight surrounding channels built to route power to the light sources. The rear end cap is designed to hold the Arduino Nano3, which controls the illumination system. Single component views, associated sections, and auxiliary views of critical design features of the sheath and housing are shown in Figs. 3–5.

The inserter sheath is constructed with a front-end outer diameter of 27.0 mm, an internal diameter of 25.5 mm, and an insertable length of 65.0 mm (Fig. 3). This maximizes comfort and reduces friction during insertion and image acquisition. The rear end of the inserter is designed for ergonomic handling and protection of the imager system, extending to approximately double the insertable length.

The front-end imager houses the polarization illumination, optical elements, and the LUCID polarized camera. The overall front housing is 82 mm in length. The front (Fig. 4, red surface) critical surface of the housing is designed with a diameter of 25.0 and 4.4 mm depth for a 0.5 mm diameter clearance interface between the housing and the inside of the inserter sheath. The surface is specified to have a minimum surface finish of 25.8±1.0  μm, equivalent to a P600 sandpaper finish. The more refined surface finish on this surface is required to have a smooth interface when inserting the imager into the inserter sheath. The rear of the housing (Fig. 4, green surface) is created to fit inside the back-end housing with a clearance fit of 0.2 mm.

The polarized illumination is achieved with 8 LEDs controlled by an Arduino board and a custom software interface. Inside the front end, slots are created for seating the eight LEDs, and pairs of LEDs are used to provide a more uniform illumination. Each slot is evenly spaced throughout the diameter of the front of the housing at 45 deg apart from each other. The slot dimension for the LED is 2.5×2.5  mm, as shown in Fig. 5. The LED has a dimension of 2×2×1.5  mm. The LEDs are manufactured by Bowerful with a 120 deg viewing angle; they are low profile 2×2  mm with emission wavelength 511±40  nm. The forward voltage for powering the LEDs is 3 to 3.3VDC, which is compatible with Arduino output. The total eight LED light intensity measured with a power meter with all LEDs illuminated is $0.65\;\frac{\mathrm{mW}}{{\mathrm{cm}}^{2}}$. Each LED slot is fitted with a channel traveling the entire length of the casing to allow for connection to the Arduino. The slot dimension for the polarizing elements is 3.5×6.5×1.0  mm (Fig. 5). Each slot is fitted with a section to fix these elements with adhesive.

**Fig. 6:**
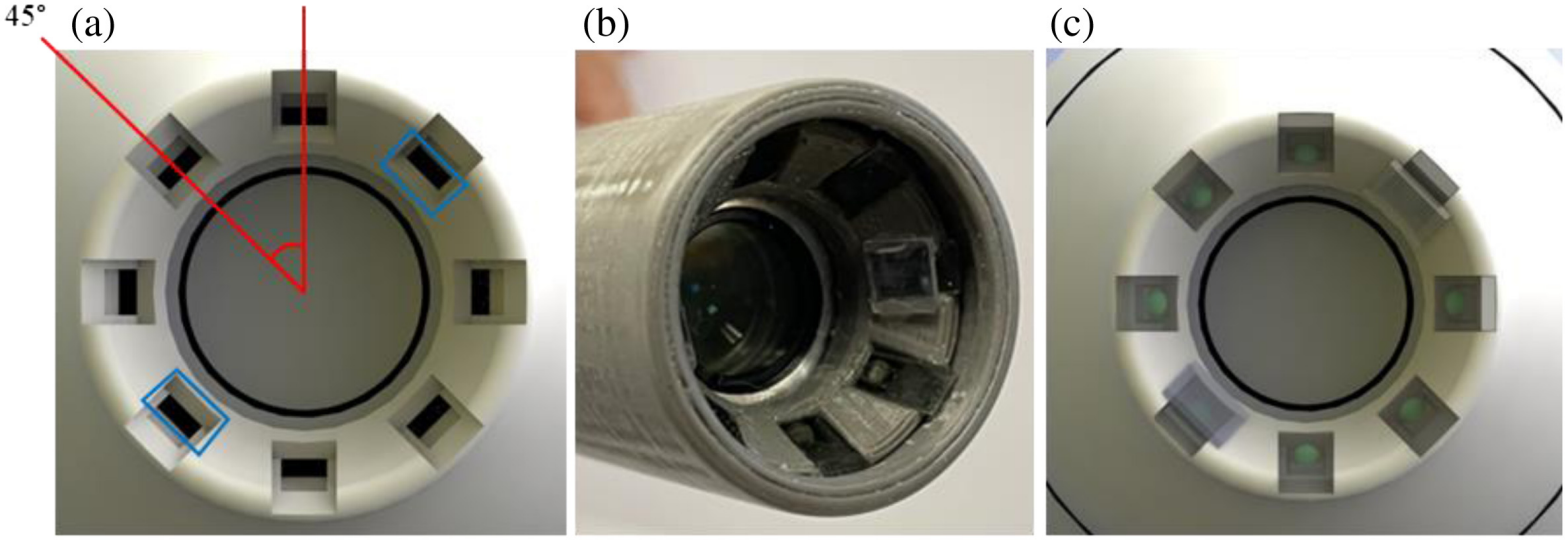
(a) Polarizer and LED pocket position at 45 deg increments, the blue rectangles indicate the position of two right circular polarizer/LED combination; (b) prototype model with fixed LEDs, polarizers, and quarter waveplates, and (c) model front view of illumination elements.

The LEDs are seated at a tilted angle along the ring illuminator face at the distal opening for the front housing assembly. The polarization elements are three linear polarizers at 0 deg, 45 deg, and 90 deg, with respect to the reference plane created by the camera illuminator and sample, and a circular polarizer (Fig. 6). Careful calibration was used to establish alignment of the polarization elements on the LED pairs.

**Fig. 7:**
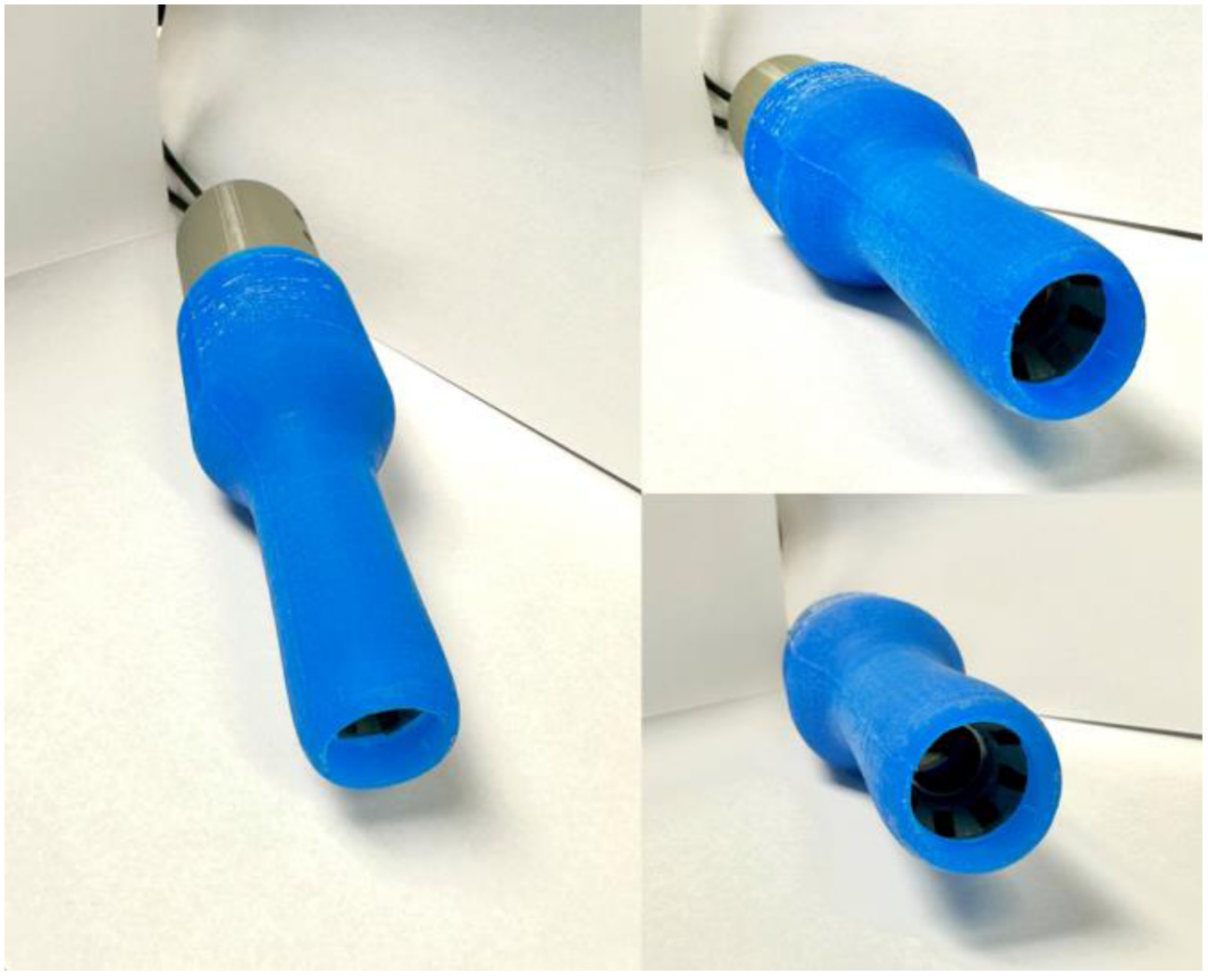
PPRIM prototype system with a flexible sheath. The main body casing is shown inserted into the flexible sheath.

The prototype system printed using PLA on a MakerBot 5th Generation Replicator Plus is shown in Fig. 7 with casted silicon casted inserter.

The camera inside the PPRIM is a Phoenix PHX050S-PC polarized camera by LUCID Vision Labs, British Columbia, Canada. The Phoenix camera has a square body with dimensions of 24×24  mm and 27.4 mm in length, with a 5 Megapixel Sony IMX250MZR CMOS sensor with a 2448×2048  pixel resolution and a maximum frame rate of 22 frames per second. The camera is mounted onto the system via a C-Mount to SM05 flange to the optical lens tube. The camera connects to a computer via an RJ45 GigE Vision ethernet connector. The camera pixels are mapped to a specific polarization state in sets of 2×2  pixel arrangements, allowing for the simultaneous capture of four linear states. The camera is controlled through a custom-made program (MATLAB^®^), allowing users to view and capture real-time live images of the cervix.

**Fig. 8:**
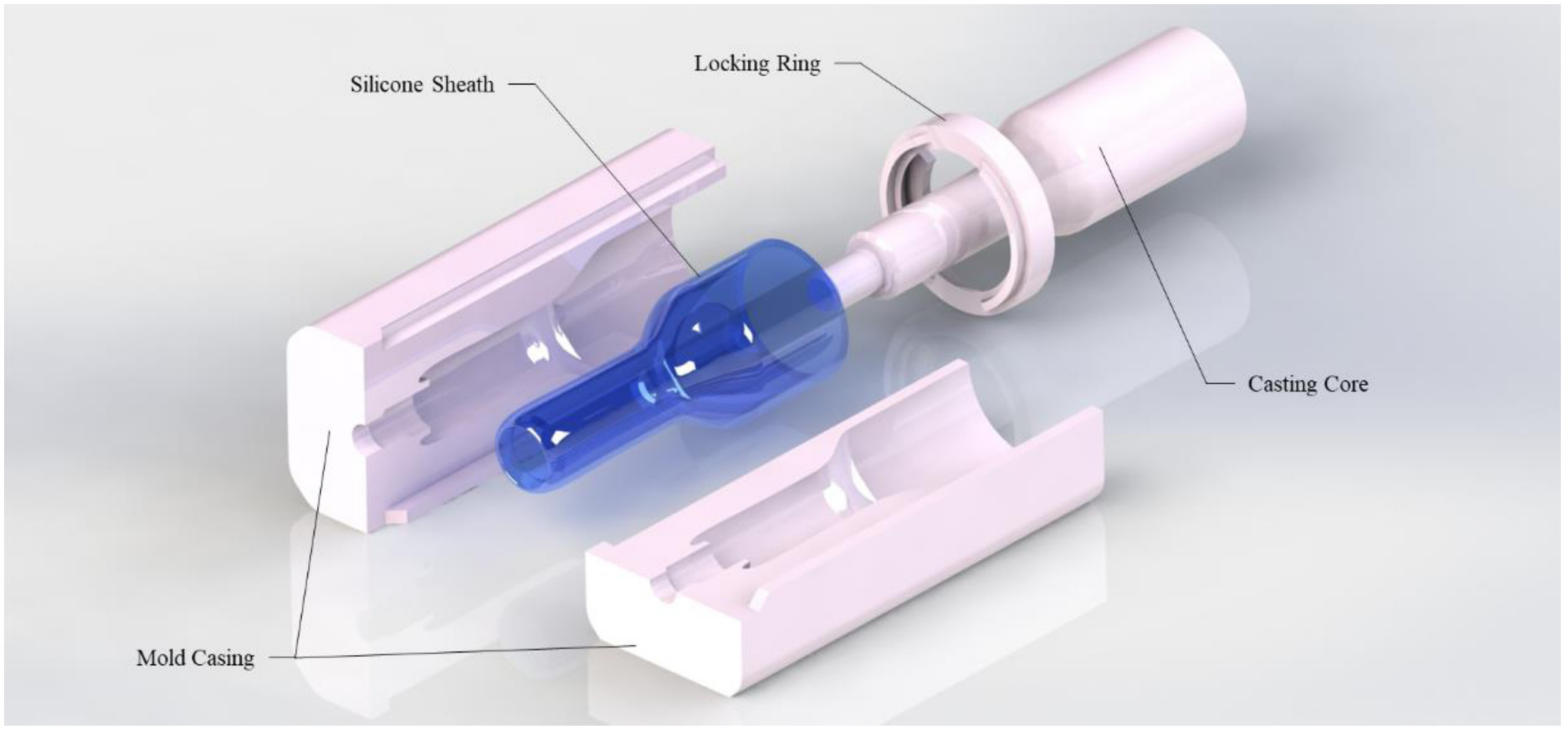
3D SolidWorks model of casting mold for silicone sheath. The four main components of the casting mold include the two-mold casting body, the internal casting core, and the locking ring.

**Fig. 9:**
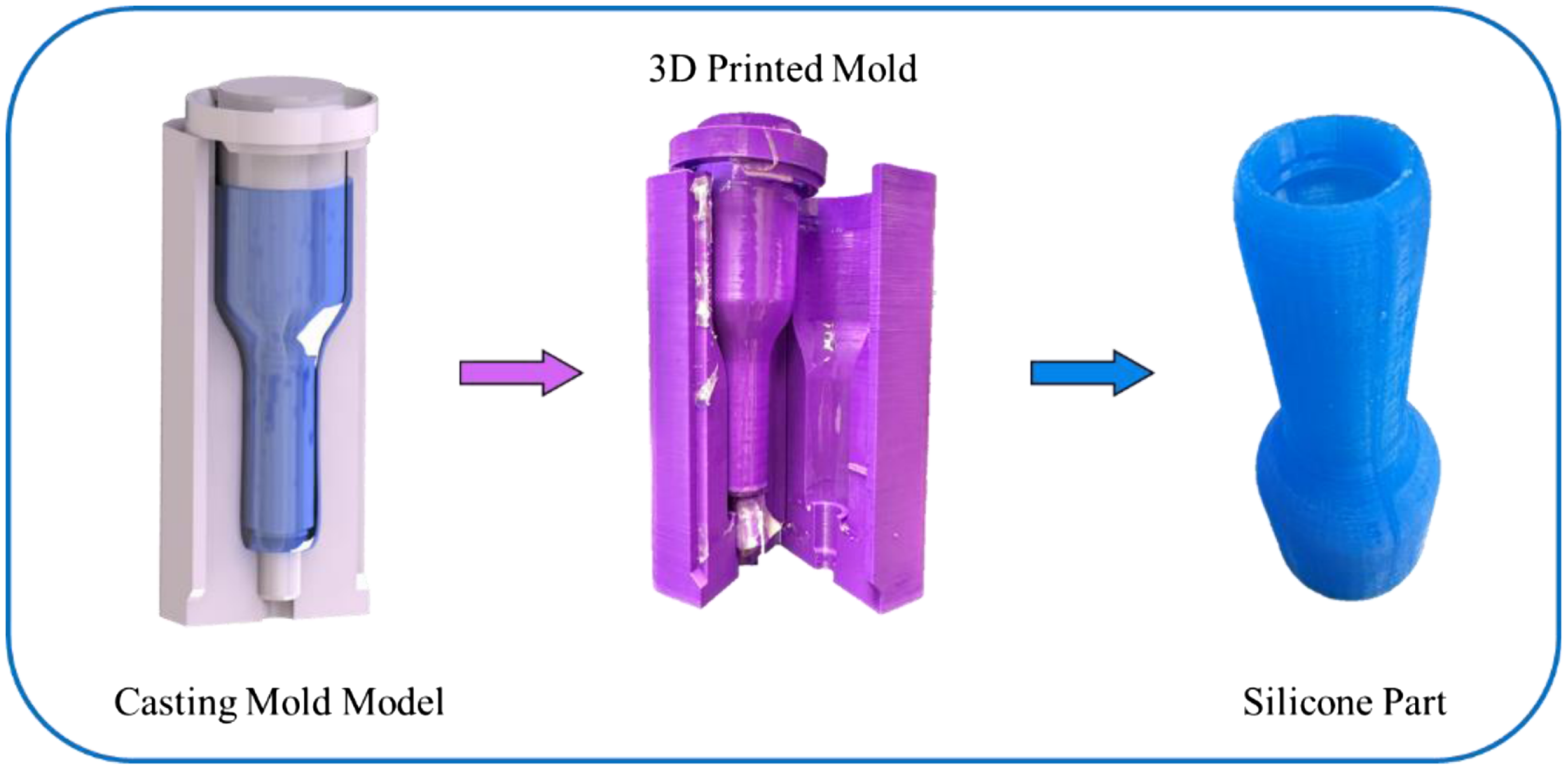
PPRIM inserter sheath silicone casting process images from casting mold model to 3D printed mold and the final silicone inserter product in blue.

The design parameters of the inserter sheath are controlled by the size of the vaginal canal and user comfort. This limits the insertable length to 65 to 70 mm and the diameter to 27 mm. The initial prototype design material for the inserter sheath is built through different 3D printed materials ranging from rigid plastic down to Shore 85A flexible rubber-like substances. For more rigid prototypes, the sheath must be post-processed to meet a maximum surface finish requirement of 18.3±1.0  μm and other processes to remove abrasions and defects from manufacturing to maximize comfort. Design iterations and testing required increasingly flexible and softer material; this resulted in the final development of a silicone-casted inserter sheath. The casting mold is created with 3D-printed PLA with a negative cavity of the sheath (Figs. 8 and 9). The mold has four different sections: two locking outer shell bodies, an internal casting core with dimensions of the front-end housing of the imager, and a locking ring to prevent tilting of the internal core. The final silicone sheath is shown in Fig. 9. A curved tip design at the head of the inserter facilitates the insertion process.

Each sheath is disinfected and cleaned using an autoclaving procedure defined by the Centers for Disease Control (CDC) for health guidelines. Autoclaving or steam-sterilization temperatures are 121°C (250°F) and 132°C (270°F).^29^ Under gravity displacement autoclave, the sheath can be sterilized at 121°C for 30 min with a 15- to 30-min drying time. The silicon sheath has a heat resistance rating of up to 232°C (450°F).

### 2.2 Optical Design

**Fig. 10:**
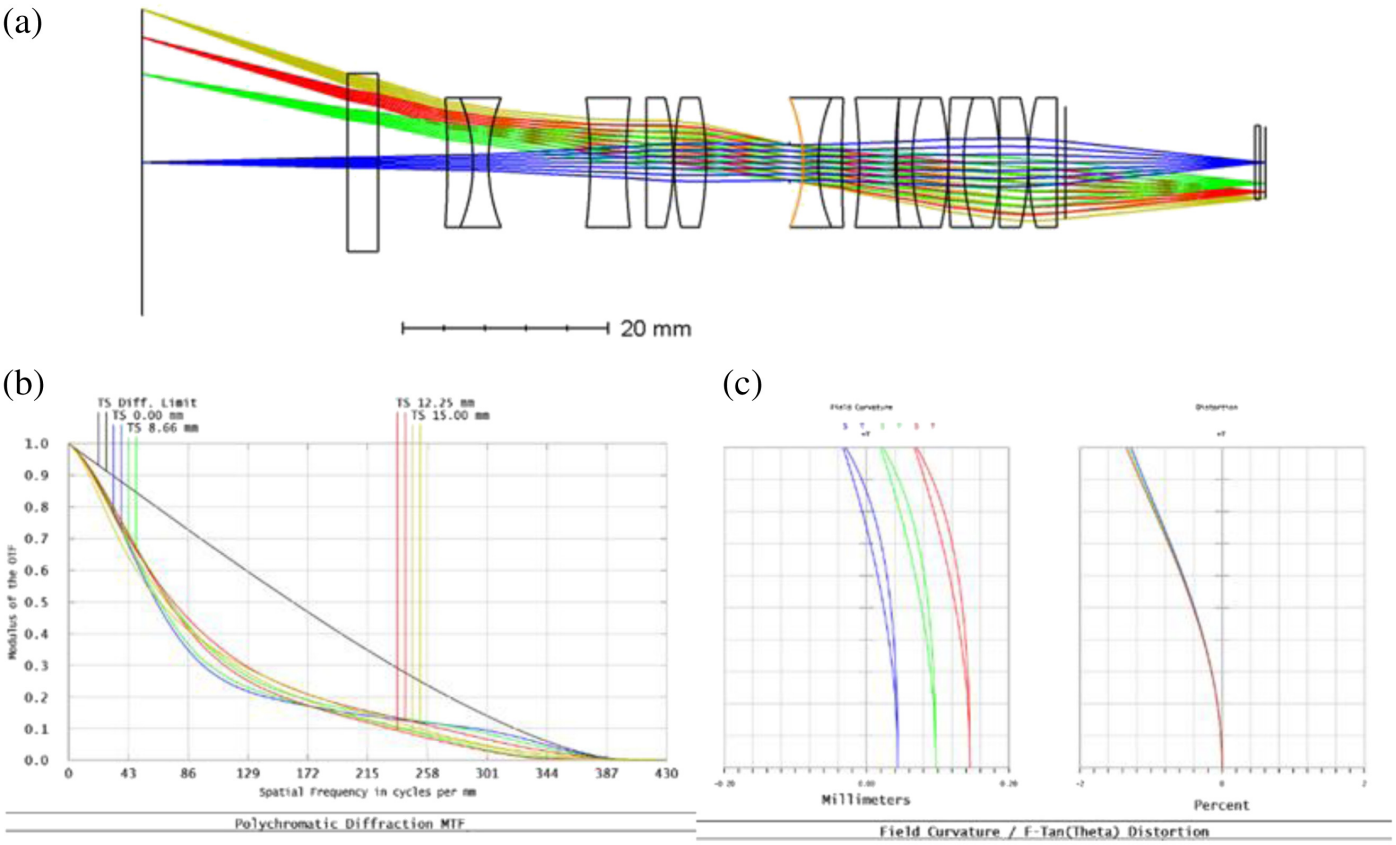
Final optical design layout with light ray traces at 0.5in stock optics. (a) Zemax simulation results, (b) polychromatic diffraction simulation, and (c) field curvature simulation.

The optical layout comprises a reverse telephoto lens positioned in front of the stop, which allows for an extended front working distance. The lenses behind the stop form a telecentric relay, similar to the tube lens in an infinity-corrected microscope system. To minimize sensitivity to alignment and fabrication errors and reduce polarization aberrations, the angle of incidence (AOI) of the optical elements is kept to a minimum. The optical train was modeled using Zemax (Fig. 10), revealing a field of view (FOV) of ∼25  mm in diameter at a working distance of 20 mm. The maximum image space F-number is 6.63, with a magnification of 4.25, whereas the maximum object space F-number is 28.17. The design aims to achieve minimal aberration of the polarization signature and is feasible using readily available lenses.

A glass window is placed before the last lens to protect the optics. The optical train spans 70 mm. The camera requires a C-mount attachment, resulting in a back working distance greater than 17.5 mm. Due to lenslets and polarizers placed directly on the camera sensor pixel, the chief ray angle is minimized for two primary reasons. First, the increased contrast ratio of the polarizers decreases with increased AOI, and second, lenslets on the pixel result in decreased signal with increased AOI.

The design of the scope follows a concept similar to that of a standard endoscope. One of the main design challenges is accommodating larger optical elements to achieve increased image size. However, the size limitation of the scope necessitates maintaining a small diameter for the optics, resulting in increased design complexity. To preserve the accuracy of the polarization state in each image, it is crucial to maintain a small AOI on all optical surfaces, effectively minimizing polarization diattenuation and retardance. The diameter of the optical train, constrained by the optics tube diameter, represents a significant limitation on the overall size of the PPRIM system.

The optical design fully uses the sensor while satisfying the required FOV of 25 mm diameter. The diameters of the optical element are restricted to less than 20 mm. The modulation transfer function plot shows near-limited diffraction performance across the entire FOV. The distortion plot demonstrates low distortion. The optical elements have a simple quarter wave MgF2 AR (magnesium fluoride anti-reflective) coating. Polarization pupil plots are performed for various polarization states; the analysis demonstrates the preservation of the polarization states. The optical elements are restricted to stock optics with a maximum diameter of 12.7 mm or 0.5 in.

### 2.3 System Control and User Interface

The PPRIM is connected to a computer through two different connections: ethernet GigE vision connect and mini-USB. The GigE vision connector interfaces with the camera, and the mini-USB connects with the Arduino Nano3. The eight LEDs are controlled through four pulse-width modulated digital pins on the Arduino. As each pin is activated for a specific polarization state illumination, two LEDs opposite each other will turn on. A custom MATLAB-based interface was built to control both imagers, Arduino board and LEDs.

The data acquisition sequence turns off all illumination, initializes the first LED pair for the first polarization state, and then the camera captures an image of the cervix. This process is repeated three additional times for three other polarization states of the PPRIM. The acquisition sequence for all four polarization states takes up to 3 s; the data are automatically saved once the imaging process is complete.

### 2.4 Data Analysis and Processing

Decomposition of the sample Mueller matrix (M) can be obtained using the Lu–Chipman polar decomposition as^30^

$$M=[\begin{array}{cccc} 1 & 0 & 0 & 0\\ 0 & d_{1}(c^{2}+s^{2}\;\cos\;R) & d_{1}cs(1-\cos\;R) & -d_{1}s\;\sin\;R\\ 0 & d_{2}cs(1-\cos\;R) & d_{2}(s^{2}+c^{2}\;\cos\;R) & d_{2}c\;\sin\;R\\ 0 & d_{3}s\;\sin\;R & -d_{3}c\;\sin\;R & d_{3}\;\cos\;R \end{array}]\;.$$ (1)

R is the scalar linear retardance, $c=\cos(2\theta_{R})$ and $s=\sin(2\theta_{R})$, where $\theta_{R}$ is the azimuth. The system captures a 3×4 Mueller matrix by combining four generated input polarization states, three linear states and one circular, and the capture linear states by the polarized camera.^30^^,^^31^ The 3×4 decomposition allows for the elimination of the circular state at the polarization state analyzer (PSA), enabling the use of a commercial polarized camera, which is limited to only capturing the linear states.^30^ The reduced 3×4 Mueller-matrix decomposition is then used to obtain depolarization, retardance, and azimuth.^31^ The AoP, linear retardance, and depolarization were calculated with the following equations:^30^

$$\theta_{R}=0.5\;\arctan(\frac{-m_{24}}{m_{34}})=\mathrm{AoP},$$ (2)

$$R=2\;\arctan(\frac{sm_{34}m_{23}-cm_{24}m_{32}}{2cs(m_{33}m_{23}+m_{22}m_{32})}),$$ (3)

$$d_{1}=-\frac{m_{24}}{s\;\sin\;R},$$ (4)

$$d_{2}=\frac{m_{34}}{c\;\sin\;R}.$$ (5)

Equations (2) and (3) can be further simplified under the assumption of isotropy of linear depolarization, where $d_{1}=d_{2}=d$

$$d=\frac{\sqrt{m_{34}^{2}+m_{24}^{2}}}{\sin\;R}.$$ (6)

The AoP from Stokes vectors and degree of linear polarization (DoLP) defined by Ref. 32 are also observed:

$$\mathrm{AoP}=\frac{1}{2}\;\arctan(\frac{S_{2}}{S_{1}}),$$ (7)

$$\mathrm{DoLP}=\frac{\sqrt{S_{1}^{2}+S_{2}^{2}}}{S_{0}},$$ (8)

where $S_{0}$, $S_{1}$, and $S_{2}$ in Eqs. (2) and (3) are the first three Stokes parameters. The Stokes vectors are calculated using the equations below:^32^

$$S_{0}=I_{0}+I_{90}=I_{45}+I_{135},$$ (9)

$$S_{1}=I_{0}-I_{90},$$ (10)

$$S_{2}=I_{45}-I_{135}.$$ (11)

## 3 Results

**Fig. 11:**
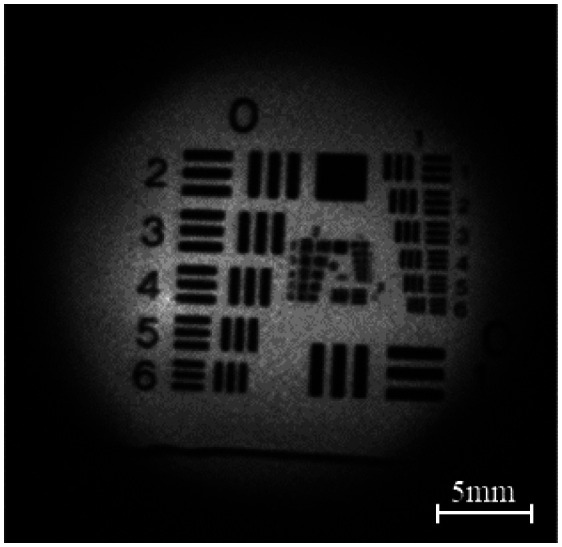
Image of a USAF target obtained with PPRIM.

**Fig. 12:**
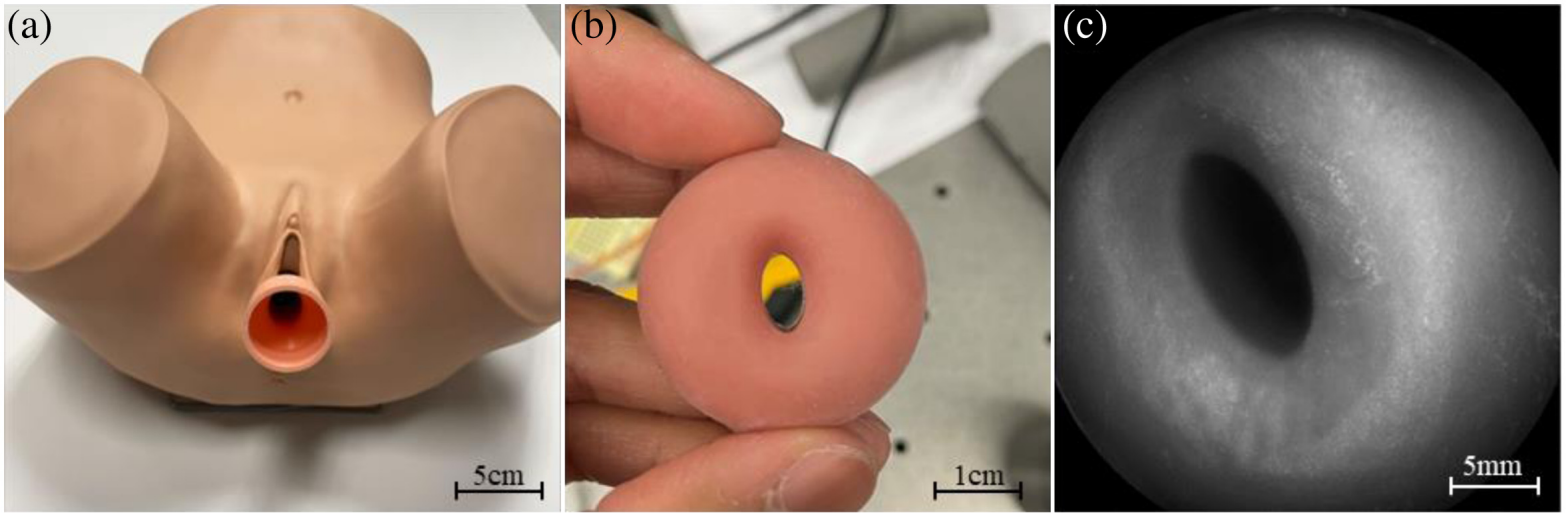
(a) ZOE anatomical model with inserter, (b) the image of silicone cervix phantom removed from the model, and (c) raw PPRIM image of cervical phantom.

Bench testing of the PPRIM system was performed on a USAF resolution target (Fig. 11). The resolution of the PPRIM is determined using a USAF target. The PPRIM was determined to have a resolution of 6.35  lp/mm and a resolution of 78.75  μm. The system meets the requirements of a 25 mm FOV and 20 mm working distance. Further bench testing was performed on a silicone gynecological training system for physicians to evaluate cervical cancer (ZOE Gynecologic light skin tone skills trainer, Gaumard). First, the sheath is inserted into the vaginal canal to act as the barrier interface between the human body and the main PPRIM imager. An example of a PPRIM image of the silicone cervix phantom is shown in Fig. 12.

**Fig. 13:**
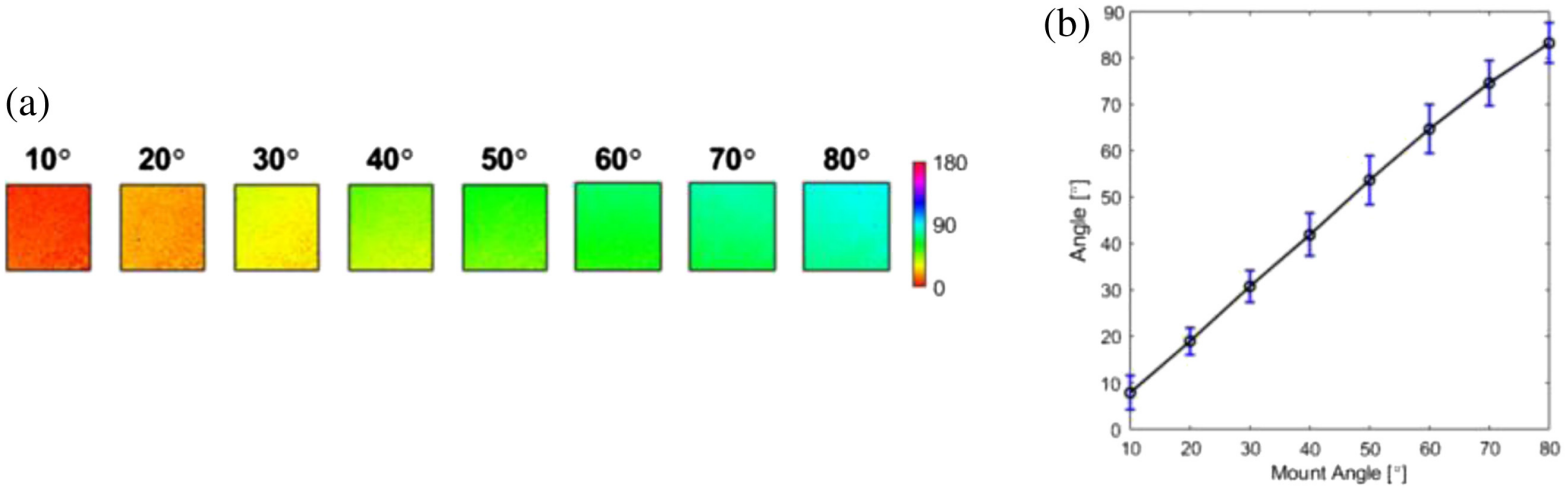
Birefringent silicone sample testing. The silicone sample is attached to a rotational stage under applied tension in a uniform direction. The stage is rotated to 80 deg at angular increments of 10 deg. (a) The azimuth angle of the sample indicates angular rotation from 10 deg (red) to 80 deg (cyan), as indicated by the color bar. (b) The plot indicates the sample’s mean angle and standard deviation at each rotational mount angle.

Birefringent samples were used to characterize the PPRIM’s performance for extracting relevant parameters, including scalar depolarization and azimuth angle. Figure 13 shows the testing of the PPRIM system on a silicone sample. The sample was secured under a rotational stage. The stage was rotated from the initial position at 10 deg to 80 deg at increments of 10 deg. The PPRIM captured images at each rotation angle of the mount. The images indicate the silicone sample’s resolved azimuth angle [Eq. (2)] at each mount position [Fig. 13(a)]. The azimuth of the sample transitions from 0 deg to 80 deg through each 10 deg incremental angle [Fig. 13(b)].

**Fig. 14:**
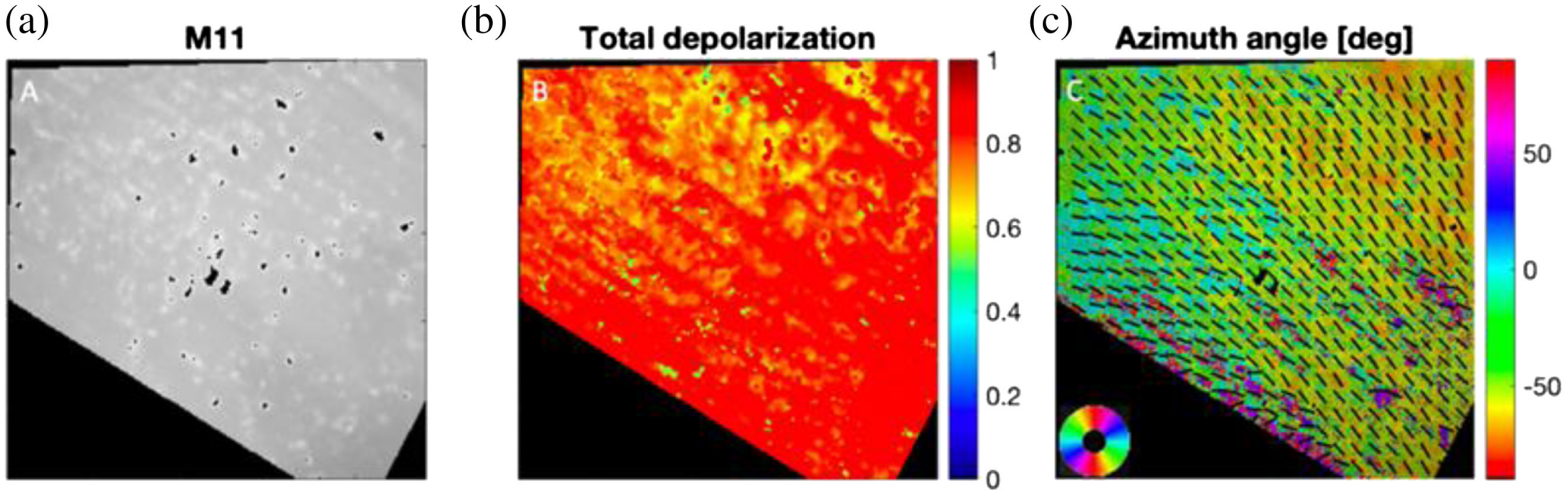
Excised birefringent Mueller-matrix decomposition. Chicken tendon sample aligned at −30  deg to the benchtop. (a) Intensity image M11, (b) total depolarization, and (c) azimuth angles. The black bars indicate the preferred alignment of the tissue within a 10-pixel range. Scale bar 5 mm.

A chicken tendon was also measured; the thin tendon fiber was arranged and secured to the benchtop at a −30  deg angle with respect to the local reference frame. Figure 14 shows the images acquired with the PPRIM. The total depolarization image of the tendon [Fig. 14(b)] shows the characteristics of highly depolarizing tissue with a median value of 0.89. Biological tissues are highly scattering media and are expected to have a high depolarization value (the highest depolarization value possible being one). Moreover, the chicken tendon oriented at −30  deg is correctly captured, Fig. 14(c); the calculated angles can be observed by the black bars representing a 10-pixel range.

**Fig. 15:**
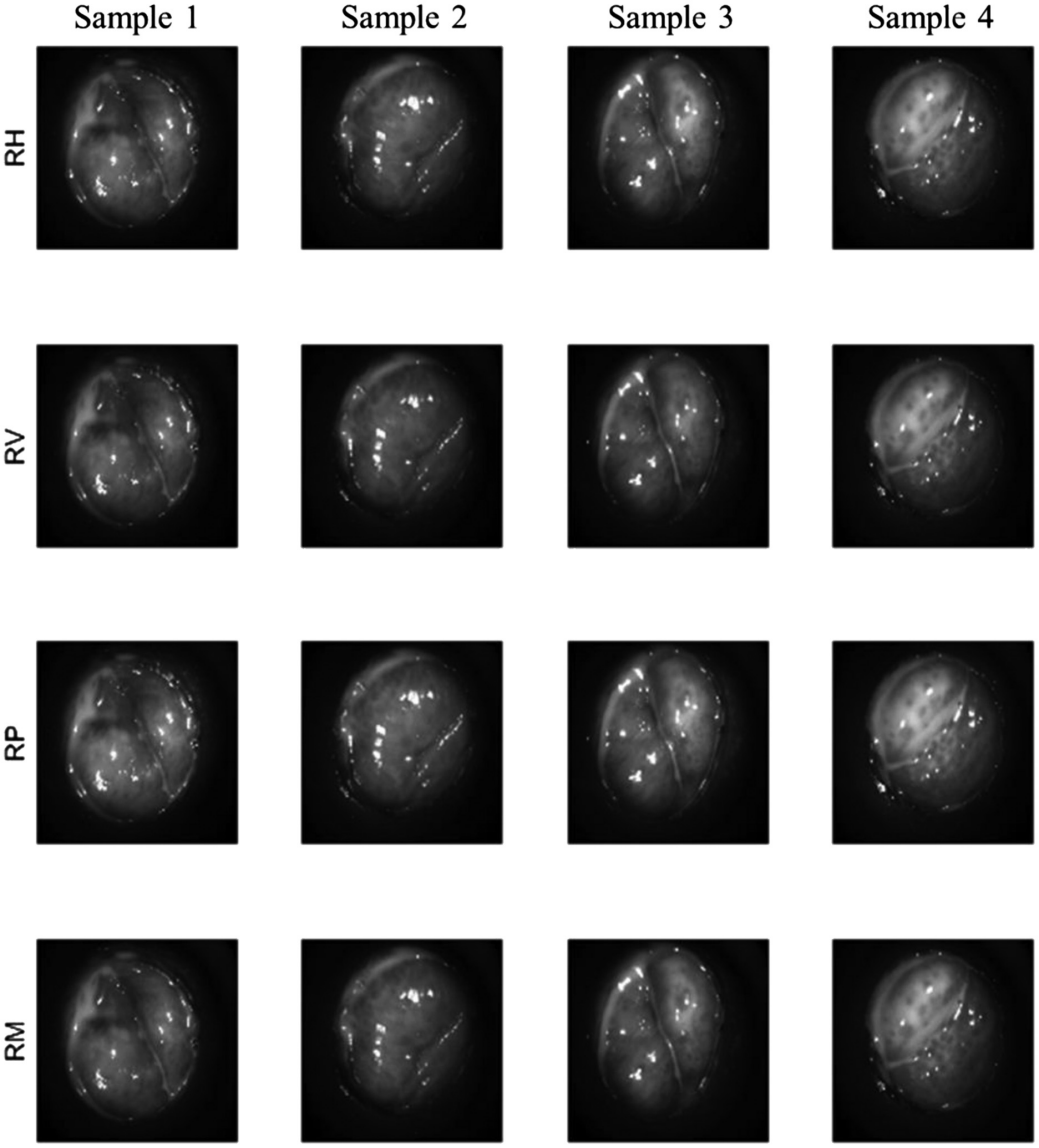
*In vivo* human image samples from PPRIM polarized camera capture. Each column image is captured simultaneously and split into the corresponding polarization image channels. All images were taken with the right circular input state illumination. The first row (RH) is the horizontal polarization state, the second row (RV) is the vertical polarization state, the third row (RP) is the positive 45 deg, and the last row (RM) is the −45  deg polarization state. The scale bar in sample 4, RM image shows 5 mm.

The PPRIM was tested on healthy volunteers with informed consent under the approval of IRB protocol IRB-21-0173-AM01 granted at Florida International University. A water-based lubricant was applied before sheath insertion for participant comfort. A digital mask was used to limit the FOV to cervical tissue, eliminating sections of the vaginal canal. Figure 15 shows four sets of images from a non-pregnant subject.

**Fig. 16:**
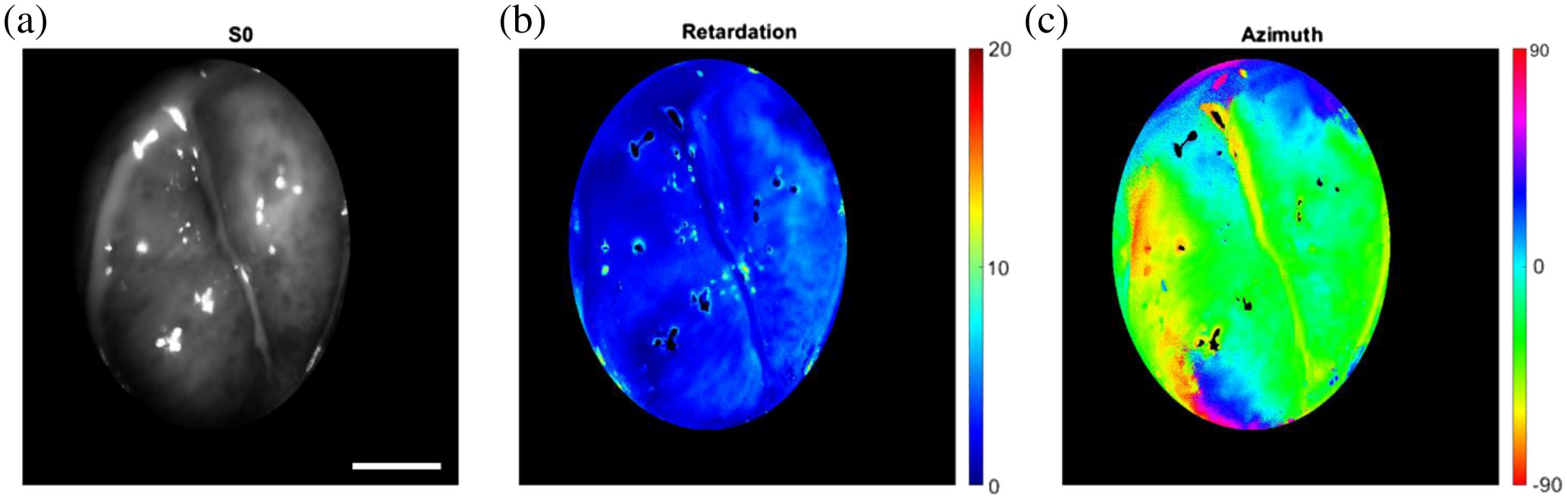
*In vivo* human cervix sample showing (a) the intensity image, M11, (b) the retardation, and (c) the azimuth orientation, as shown by the azimuth angle. Scale bar 5 mm.

In Fig. 16, we show typical results obtained from a healthy volunteer. First, the element of the Stokes vector (S0) shows the total intensity of the cervix captured by the PPRIM. Using the 3×4 decomposition of the Mueller matrix, the total retardation and azimuth angle of the cervical tissue are calculated.

## 4 Discussion

PPRIM underwent several tests for both usage and image quality. Under the original specification for a speculum-free system, the silicon casted insert sleeve, by far, outperformed all other materials and different manufacturing processes. The significant advantage of the silicon sleeve is an easier and more effective sterilization process using a steam autoclave and increased comfort for the user. The malleability and flexibility of silicone allow it to conform to the shape of the casting mold. This means that the front-end housing of the device body will provide the primary support during insertion. This remains within the design parameters of the PPRIM and accounts for vaginal wall forces.^33^ The insertable process was described anecdotally by volunteers as comfortable and much preferable to using a speculum. In future clinical testing and with more participants, a more systematic approach will be developed to obtain an evaluation of the PPRIM by both the care providers and the patients.

The PPRIM was able to capture images of the full cervix and portions of the vaginal walls; values of depolarization and retardation obtained with the PPRIM are in the same range as the one obtained with a previous system developed by our group utilizing a speculum and a colposcope.

PPRIM testing on known birefringent samples, illustrated in Figs. 15 and 16, has shown the capabilities of the PPRIM in resolving angular information using polarization techniques. The calculations of the azimuth angle of the birefringent samples follow the methods indicated by Novikova and Ramella-Roman.^30^ The PPRIM is designed to operate and analyze images using a 3×4 Mueller-matrix decomposition method;^31^ the 3×4 eliminates the requirements for the PSA to include the acquisition of the right circular state, which is only relevant to the last column of the Mueller matrix [Eq. (1)]. This allows for the simplification of the PSA and the use of the small form factor camera such as the current LUCID camera. The 3×4 Mueller-matrix decomposition, as demonstrated by Gonzalez et al.,^31^ provides sufficient information to acquire the sample’s azimuth, retardation, and depolarization properties [Eqs. (2), (3), and (6)].

A 3×4 Mueller matrix^31^ can be captured at an acquisition rate of 0.56 s per image set. This equates to 1.80 frames per second. This sequence includes four LED illumination on/off cycles at 0.04 s for a total of 0.16 s and four images (12-bit, 2440  pixels×2444  pixels) at 0.1 s per image. At 8 bit (2440  pixels×2444  pixels), a complete 3×4 Mueller-matrix capture can be achieved in 0.36 s, sacrificing image size the speed can be improved. For calculation based only on a right circular input state to obtain AoP,^30^ this system can capture a single snapshot at 22 fps, which is the limit of the LUCID polarized camera. Other polarized cameras can capture at a high frame rate, up to 35 fps.^34^ However, this is at the cost of a large form factor, which would render the PPRIM less portable.

## 5 Conclusions

The combination of Mueller-matrix imagery into a portable imaging system holds strong potential to aid in the early diagnosis and assistance for those at risk of preterm birth. We have developed the PPRIM device designed around ease of use and minimizing discomfort for women undergoing this type of testing. This device eliminates the need for a speculum used in standard clinical cervical examination. It can allow the system to be self-inserted, and future studies will focus on point-of-care testing. The PPRIM shows good image quality when used to evaluate a standard USAF 1951 resolution target, a model cervical phantom, and *in vivo* testing. Moreover, the polarimetric results shown in this paper compare well to previously published data on similar samples. This system will be helpful in diminishing patient attrition during studies of polarization imaging of the human cervix. Studies utilizing this modality for cervical cancer diagnosis could also benefit from this apparatus. Finally, this tool’s ease of use and versatility make it very suitable for *in vivo* polarization-sensitive imaging beyond the cervix. It could be integrated into multiple diagnostic applications focusing on women’s health. With the current state of healthcare trends increasing the value of telemedicine and remote care, the PPRIM can provide increased access and communication between healthcare providers and women with new information channels previously unavailable.

## Data Availability

The datasets used and/or analyzed during the current study available from the corresponding author on reasonable request.
